# Anti-hyperglycemic activity of *Centella asiatica* is partly mediated by carbohydrase inhibition and glucose-fiber binding

**DOI:** 10.1186/1472-6882-14-31

**Published:** 2014-01-18

**Authors:** Ashraf Ul Kabir, Mehdi Bin Samad, Ninadh Malrina D’Costa, Farjana Akhter, Arif Ahmed, JMA Hannan

**Affiliations:** 1 Department of Pharmacy, North South University, Dhaka, Bangladesh

## Abstract

**Background:**

*Centella asiatica* (*C. asiatica*) was previously reported to have anti-hyperglycemic effects in animal diabetic model rats. However, its activity on organ and tissue level remains unstudied. Our study aims at exploring the possible effects, *C. asiatica* extract and insoluble fiber has on carbohydrate absorption, insulin secretion, insulin sensitivity and glucose utilization.

**Methods:**

For primary evaluation of anti-hyperglycemic activity, we measured Fasting Blood Glucose and performed Glucose Tolerance Test, in type 2 diabetic rats. To further study the pancreatic effect and glucose utilization, plasma insulin concentration, insulin secreted from isolated rat islets and liver glycogen were assayed. Effect on carbohydrate break down was assayed using intestinal disaccharidase enzyme, α-amylase inhibition assays and Six-Segment study of the GI tract. Effect of *C. asiatica* on glucose absorption was studied by an in-situ, perfused, intestinal model in rats and by glucose-fiber binding assay. Gastrointestinal motility was seen by a BaSO_4_ milk traverse test. Additionally, a complete lipid profile assay, after a chronic study, was conducted.

**Results:**

*C. asiatica* showed no significant change in insulin secretion in-vivo and in isolated rat islets. Additionally, no effect of the extract was seen on liver glycogen deposition. Retarded glucose absorption was seen in the in-situ perfused rat intestinal model at a dose. The extract was also found to inhibit action of both intestinal disaccharidase and α-amylase. This was confirmed, yet again, via the Six Segment study, where sucrose digestion was found to be inhibited throughout the length of the GI Tract. Significant glucose-fiber binding was demonstrated in the in-vitro models. During the chronic study, body mass of *C. asiatica* treated Type 2 diabetic rats returned to normal and their polydipsic and polyphagic conditions were also improved. Chronic treatment of *C. asiatica* also improved subject’s lipid profile.

**Conclusion:**

A combination of in-vitro, in-vivo and in-situ tests confirmed the anti-hyperglycemic activity of *C. asiatica* and its tissue level mechanism. Further study is required to fully elucidate the effect this extract or the active compounds have on the individual glucose transporters and the precise mechanism of glucose-fiber binding.

## Background

In the last few decades, diabetes has established its position as one of the world’s predominant endocrine disorder [1]. The number of people to be affected by this disease by the year 2000 was estimated to be 171 million, by World Health Organization [1]. Diabetes, by nature, cannot be completely cured rather it has to be kept under tight control [2]. This control might be achieved, by modifying lifestyle, medications, diet, or a combination of all of these [3]. Many of the drugs currently in use, are expensive and have adverse effects which, in tandem, makes diabetes management even more difficult [4]. Complementary and Alternative Medicines (CAM) therefore have a large room to bring about improvements in the current practices of diabetes management. CAM is rapidly growing popular throughout the world [5]. Furthermore, CAM therapy is relatively cheaper than synthetic, patented drugs in the perspective of developing countries. A large segment of the population in these countries are traditionally reliant on CAMs for managing a multitude of disorders including, diabetes [6]. However, evidences regarding efficacy of these therapies are still sparse and their mechanism of action is often unclear [7]. *Centella asiatica* is a widely used traditional remedy in both Africa and India. The water extract of the whole plant is used by traditional healers in Bukoba district, Tanzania for the management of both Type I and Type 2 diabetes [8]. The plant has found similar use in Trivandrum and Kanyakumari Districts in India. Studies have found *C. asiatica* possessing significant hypoglycemic activity in glucose tolerance test in rabbits. It was also found not to cause hypoglycemia in fasted rabbits compared to the standard tolbutamide [8]. The ethanolic extract showed an increased glycogen content in the liver, comparable to the glibenclamide standard [9]. Addtionally, the extract showed lowered serum cholesterol and total lipid level [9,10]. Methanolic extract was found to be more effective than ethanolic extract in lowering blood glucose [9]. However, may aspects of basic mechanism of action of *C. asiatica* still remain unclear to date. The aim of the current study is to paint a comprehensive picture of effects *C. asiatica* on sucrose breakdown and glucose absorption, insulin release, and intestinal enzyme functions. Our study helps to identify the particular organ or organ system responsible for the previously seen hypoglycemic activity of this plant.

## Methods

### Plant collection and processing

*C. asiatica* was collected as whole plant from University Ayurvedic Research Centre (UARC), Jahangirnagar University, Dhaka, Bangladesh. The plant was identified by a botanical taxonomist prior to further processing and a voucher specimen was deposited at the National Hebarium at Mirpur, Dhaka, Bangladesh. The whole plant was cleaned off of dirt and other debris and then thoroughly washed under running tap water. The plant was then air-dried in an oven at 40°C and milled into a fine powder. 100gm of this powder was then dissolved in 1 L ethanol, and shaken in an orbital shaker (550 rpm for 48 hrs). The mixture was then filtered using a fine muslin cloth to remove the coarse insoluble particles. The fine particles were forced to sediment by centrifugation (1500 rpm for 10mins). The supernatant was carefully pipetted out and was further filtered using a Whatman filterpaper. The filtrate was then concentrated by vacuum evaporation using the Soxhlet apparatus (Electrothermal™ Soxhlet extractor, UK). The concentrate was left in a refrigerator for 7 days to remove further water, converting it into a gummy substance. This then underwent freeze drying at -55°C to obtain a fine powder. The fine powder extract was kept in Scott bottles along with silica gel sachets (desiccant) until further use.

### Animal handling

Both the healthy and Type 2 diabetic rats (Long Evan type) were bred in the animal house of the Department of Pharmacy Practice, North South university. The healthy rats weighted about 180-220 gm while the Type 2 diabetic rats weighed about 160-180 gm at the time of the experiment. All test animals were kept in the North South University Animal house at an ambient temperature of 22 ± 5°C and humidity of 50-70%. 12 hrs day-night cycle was maintained to avoid fluctuations in the circadian rhythm. Standard rat pellets and filtered drinking water were made available to the test animals ad libitum throughout the experiment apart from the period of fasting prior to certain tests. During fasting only water was given. During most experimental period, the rats were kept in translucent plastic cages with wood shavings provided as bedding. Animals undergoing fasting were placed in grilled bottomed cages, with no bedding, to prevent corpophagy. The designed experimental protocol was designed and subsequently approved by the Ethics Committee on Animal Research, North South University, following the “Revised guide for the care and use of laboratory animals by American Physiological Society” [11].

### Diabetes induction

Type 2 diabetes was induced in the rats by an intraperitoneal injection of streptozotocin (STZ) in citrate buffer solution at a dose of 90 mg/kg. Rat new-borns, less than of 48 hrs age weighing 7 gm were chosen for the procedure. Experiments were conducted three months after the STZ injection. The type 2 diabetic rats were selected for the experiment after conducting an oral glucose tolerance test (OGTT) and only the diabetic model rats with blood glucose levels of 8–12 mmol/L under fasting conditions were selected for the experiments [12].

### Acute effects of ethanolic extracts of *C. asiatica* on glucose homeostasis

To evaluate effects on fasting blood glucose, the *C. asiatica* extract (250 mg/kg, 500 mg/kg and 1000 mg/kg) was suspended in distilled water and orally administered to 12 h fasted rats. The control animals received an equal volume of distilled water.

Effects on glucose tolerance were similarly evaluated by administration of *C. asiatica* extracts together with glucose (2.5 g/10 ml per kg body weight) after a fasting period of 12 h. Control group received only glucose solution.

In either cases blood was collected from the tail vein, serum separated by centrifugation and stored at -22°C until further analysis. Blood glucose was analysed by GOD-PAP method [13] (glucose kit, Randox™, UK).

To evaluate chronic effects of *C. asiatica*, type 2 diabetic rats were given extract at 250, 500, 1000 mg/kg doses by gavage, twice daily for 28 d. Control rats were similarly administered water alone (10 ml/kg body weight). Blood samples were collected from the cut tip of the tail at the times indicated in the figures. Serum was separated by centrifugation, stored and analysed as mentioned above.

### Effects of *C. asiatica* on plasma Insulin

Blood was drawn from Type 2 diabetic rats, 1 hr after administration of *C. asiatica*. The amount of insulin released from the pancreas in-vivo, was determined using, Rat Insulin ELISA Kit (Crystal Chem™, USA).

### Effects of *C. asiatica* on Insulin secretion from isolated pancreatic Islets

Pancreatic islets were isolated by collagenase digestion with minor modifications as previously described [14]. The amount of insulin released from the isolated islets was determined by, Rat Insulin ELISA Kit (Crystal Chem™, USA).

### Effects of *C. asiatica* on liver glycogen content

Breifly, the liver was weighed and finely homogenized with 20 ml of 5% trichloroacetic acid (TCA). The proteins precipitated, which was filtered off, and the clear filtrate was analysed for glycogen. The liver glycogen content was determined following the anthrone method as described previously [15].

### Effects of *C. asiatica* on intestinal glucose absorption

An in-situ intestinal perfusion technique [16] was used to determine the effect of *C. asiatica* intestinal absorption of glucose in 36 h fasted non-diabetic rats anaesthetized using Ketamine (80 mg/kg). Ethanol extract of *C. asiatica* (5 mg/mL, 10 mg/ml, 20 mg/mL equivalent to 0.25 mg/kg, 0. 5 g/kg, 1 g/kg) was suspended in Krebs Ringer buffer, along with glucose (54 g/l). These were passed through rat pyloris via a butterfly cannula and the perfusate collected by means of a tube inserted at the end of ileum. The control group was perfused with Krebs Ringer buffer along with glucose only. Perfusion was carried out at a rate of 0. 5 ml/min for 30 min at 37°C. The results were presented as percentage of absorbed glucose, calculated from the percentage change in the amount of glucose in solution before and after the perfusion.

### Effects of *C. asiatica* on sucrose absorption from the gut

The effect of *C. asiatica* on sucrose absorption from gastroinstestinal was assayed by determining the unabsorbed sucrose content following oral sucrose load by Six-Segment Study as described by Hannan et al. [17]. 12 h fasted, type 2 diabetic rats were administered 50% sucrose solution per oral (2.5 g/kg body mass) along with three doses of *C. asiatica* (250 mg/kg, 500 mg/kg, 1000 mg/kg) and equal volume of water for control. Blood was sampled at the following time intervals, 30, 60, 120 and 240 min, after sucrose load for the quantification of blood glucose. At these time intervals, some of the rats were sacrificed for determining unabsorbed sucrose contents of the GI tract. The GI tract was excised and separated into six segments: the stomach, the upper 20 cm, middle and lower 20 cm of the small intestine, the caecum and the large intestine. Each segment was rinsed with acidified ice-cold saline followed by centrifugation at 3000 rpm (1000 g) for 10 min. The supernatant was pipette out and boiled for 2 h, in sulphuric acid, to hydrolyse the sucrose. The sulfuric acid was later neutralized by NaOH solution. Both plasma glucose concentration, and the amount of glucose released from residual sucrose in the GI tract was determined. The GI sucrose content was calculated from the amount of liberated glucose [18].

### Effects of *C. asiatica* on gut motility

GI motility was determined by means of BaSO_4_ milk following the previously described method of Chattarjee [19]. BaSO_4_ milk was prepared by mixing BaSO_4_ as 10% (w/v) in 0.5% carboxy methyl cellulose to form a suspension. The ethanol extract was administered per oral, 1 hr before the oral administration of BaSO_4_ milk. Control group was administered distilled water only (10 ml/kg). Rats belonging to all groups were sacrificed 15 mins after BaSO_4_ administration. The distance travelled by BaSO_4_ milk was measured, and represented as a percentage of total length of the small intestine (from pylorus to ileocaecal junction).

### Effects of *C. asiatica* on intestinal disaccharidase enzyme activity

The assay was conducted following the procedure as described previously by Hannan et al. [20]. The ethanol extract of *C. asiatica* (250, 500 and 1000 mg/kg) was administered by gastric gavage to 20 hrs fasted non-diabetic rats. After 60mins, the rats were sacrificed and the small intestine was isolated, cut longitudinally, rinsed with ice-cold saline and homogenized in 10 ml saline (0.9% NaCl). Aliquots of homogenate were incubated with 40 mM-sucrose at 37°C for 60 min. The amount of protein was determined by DC™ Protein Kit (Bio Rad, USA). Disaccharidase activity was determined from the glucose concentration converted from sucrose as μmol/mg protein/h.

### Effect of *C. asiatica* on body mass, food and water intake of type 2 diabetic rats

The rats kept for chronic study were provided with sufficient amounts of food and water for one day. At the end of the day, the mass of food and volume of water intake was recorded. The change in body mass of the rats was also monitored at periods as shown in the graph.

### Effects of *C. Asiatica* on organ weight ratio of liver and pancreas

The animals used on the chronic study were sacrificed by cervical dislocation at the end of the study period and the liver and pancreas were excised. They were cleaned of fats, and were kept moist at all times keeping them in normal saline (0.9% of NaCl). The wet mass of the organs were immediately weighed using a digital balance. The weight of the pancreas was expressed as mg/100 gm of body weight while the weight of the live was represented as gm/100 gm of body weight, as shown in the graphs.

### Chronic effects of ethanolic extracts of *C. asiatica* on serum lipid profile of type 2 diabetic model rats

To assess chronic effects of *C. asiatica*, type 2 diabetic model rats were ethanol extract at three dosages (250 mg/kg, 500 mg/kg and 1000 mg/kg) by gastric gavages, twice daily for 28 d. Control rats were administered only distilled water of similar volume. Blood samples were collected from the tail vein, at times, indicated in the graphs. Serum was separated by centrifugation and stored at 22°C until further analysis.

### Effect of *C. asiatica* on jejunal nutrient absorption by glucose dialysis-tube retardation assay

Dry, precut dialysis sacs (inflated diam. approx. 16 mm, length = 30 cm, Sigma Aldrich™, USA) were soaked in 1 g sodium azide/L. The bag was loaded with 6 mL sodium azide (1 gm/L) and 36 mg glucose alone (the control sac) or after addition of fine powder of *C. asiatica*. The dry fibrous powder was wetted by an aqueous solution of sodium azide (1 g/L) for 14 h prior to the experiment. The sacs were closed at the ends and hung in a solution of 100 mL of sodium azide (1 g/L) and then placed in a stirred bath at 37°C for 1 hr. At 30 and 60mins time interval, 2 mL of the dialysate was analyzed for glucose by the GOD-PAP method as previously described.

The effect of fiber on nutrient absorption was indicated by the glucose dialysis retardation index:

$$-\left(\frac{\mathrm{Total}\;\mathrm{glucose}\;\mathrm{diffused}\;\mathrm{from}\;\mathrm{sac}\;\mathrm{containing}\;\mathrm{fiber}\times 100}{\mathrm{Total}\;\mathrm{glucose}\;\mathrm{diffused}\;\mathrm{from}\;\mathrm{sac}\;\mathrm{containing}\;\mathrm{no}\;\mathrm{fiber}\;\mathrm{present}}\right)$$

### Effect of *C. asiatica* on α-amylase activity

The effects of *C. asiatica* powder on starch digestibility was determined as a function of time in a fiber-enzyme-starch mixture system using a dialysis membrane with a cutoff molecular weight of 12,000 da (inflated diam. approx. 16 mm, length = 30 cm, Sigma Aldrich™, USA) as previously described with minor modifications [15]. A solution was prepared by mixing 0.2 g of powdered *C. asiatica* and 0.04 g α-amylase (obtained from human saliva, Sigma Aldrich™ USA) in 10 ml of potato starch solution (4 g/100 ml) was dialyzed in 200 ml deionised water at 37°C. Following the incubation period, 10, 30, 60, and 120 min, glucose concentration in the dialysate solution was assayed using the GOD-PAP method as described previously. The control was run without the addition of powder.

### Determination of glucose-adsorption capacity

The assay was conducted following the procedure by Ou et al. [21], where the glucose-adsorption ability (mM/mol/gm) was measured by mixing 1 g of insoluble plant powder or Carboxymethyl cellulose (CMC) with 100 mL of glucose solution at a constant temperature of 37°C for 6 hrs. This was then followed by centrifugation at 3500 rpm for 15 min. Glucose concentration in the supernatant was assayed using GOD-PAP method as previously described.

### Statistical analysis

Statistical tests were conducted using Statistical Package for Social Science Software (SPSS) ver. 20 (IBM, Inc., Chicago, IL, USA). Results are presented as means ± SEM. Data from experimental groups were compared using unpaired Student’s t test and the Mann-Whitney U test, as required. Experiments with data being collected at several time intervals, were analyzed using repeated measures ANOVA followed by Bonferroni adjustment ensuring an error margin within ≤5%. One-way ANOVA was carried out and pair-wise comparisons were made with the control group using Dunnett’s test to maintain an acceptable error margin of 5%. A two-tailed *P* value of <0.05 was considered statistically significant.

## Results

### Acute and chronic effects of *C. asiatica* on glucose homeostasis

**Figure 1:**
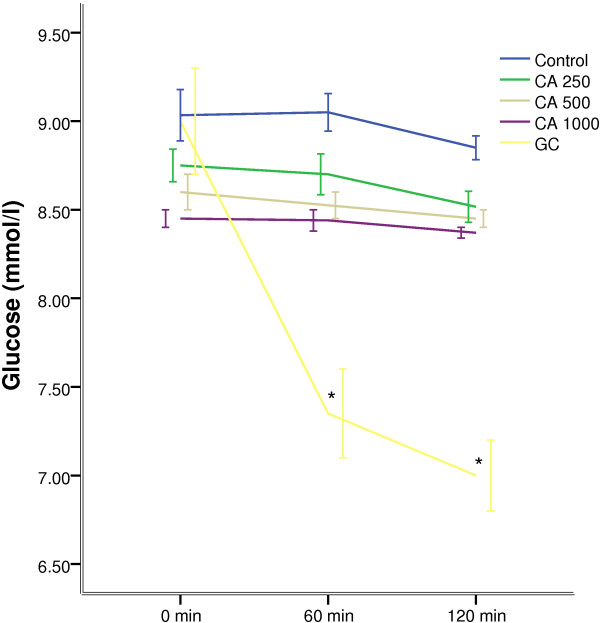
**Effects of ethanol extract of *C. asiatica *(CA) on fasting blood glucose level in type 2 diabetic rats.** Values are means and standard deviations represented by vertical bars (n = 10). Fasted rats were given ethanol extract of *C. asiatica* (250 mg/kg, 500 mg/kg, and 1000 mg/kg body weight) or Glibenclamide (GC) (0.5 mg/Kg) or only water (control) by oral administration. Mean values marked with an asterisk (*) were significantly different from those of respective control rats (p < 0.05) (derived from repeated-measures ANOVA and adjusted using Bonferroni correction).

**Figure 2:**
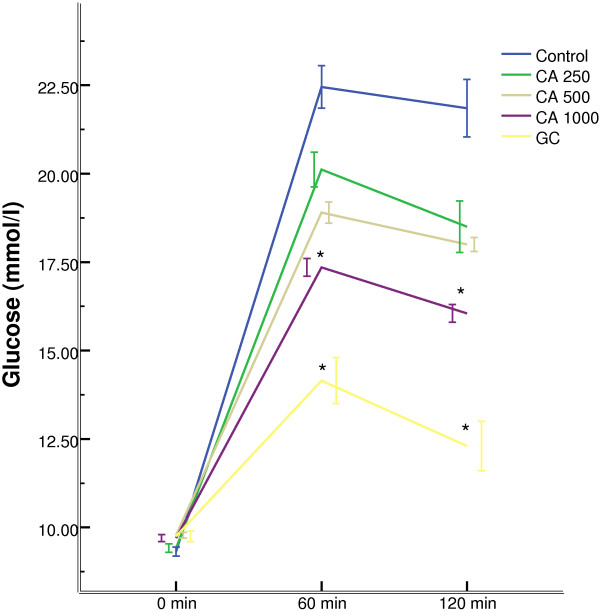
**Effects of ethanol extract of *C. asiatica *(CA) on glucose tolerance in type 2 diabetic rats.** Values are means and standard deviations represented by vertical bars (n = 11). Fasted rats were given ethanol extract of *C. asiatica* (250 mg/kg, 500 mg/kg, and 1000 mg/kg body weight) or Glibenclamide (GC) (0.5 mg/Kg) or only water (control) by oral administration with glucose (2.5 g/kg body weight). Mean values marked with an asterisk (*) were significantly different from those of respective control rats (p < 0.05). (Derived from repeated-measures ANOVA and adjusted using Bonferroni correction).

**Figure 3:**
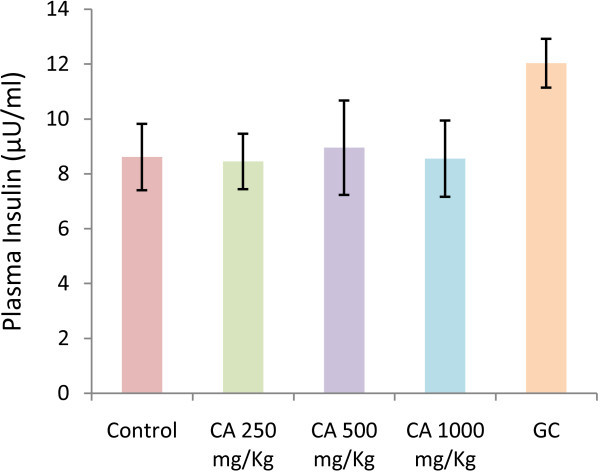
**Effects of ethanol extract of *C. asiatica *(CA) on plasma insulin level in type 2 diabetic rats.** Values are means and standard deviations represented by vertical bars (n = 8). Rats were given ethanol extract of *C. asiatica* (250 mg/kg, 500 mg/kg, and 1000 mg/kg body weight) or Glibenclamide (GC) (0.5 mg/Kg) or only water (control) by oral administration. Mean values marked with an asterisk (*) were significantly different from those of respective control rats (p < 0.05) (derived from repeated-measures ANOVA and adjusted using Bonferroni correction).

Oral administration of *C. asiatica*, at any doses, did not alter the hyperglycaemic condition of fasted type 2 diabetic rats (Figure 1). The extract, at 1000 mg/Kg dose, improved glucose homeostasis at 60 min and 120 min, when administered along with glucose load (p < 0.05; Figure 2). However, the extract did not show any effect on plasma insulin level (Figure 3).

**Figure 4:**
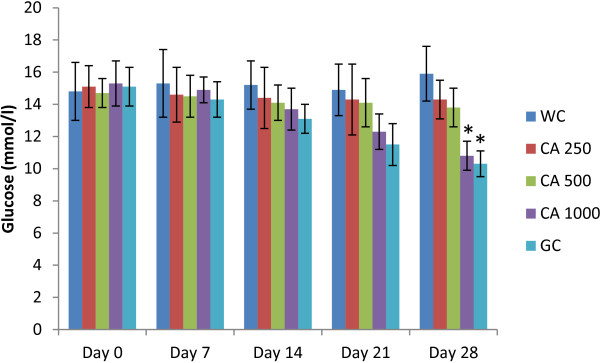
**Effects of ethanol extract of *C. asiatica *(CA) on fasting serum glucose level in type 2 diabetic rats after 28 days of feeding.** Values are means and standard deviations represented by vertical bars (n = 10). Fasted rats were given ethanol extract of *C. asiatica* (250 mg/kg, 500 mg/kg, and 1000 mg/kg body weight) or Glibenclamide (GC) (0.5 mg/Kg) or only water (water control, WC) by oral administration for a period of 28 days. Mean values marked with an asterisk (*) were significantly different from those of respective control rats (p < 0.05) (derived from repeated-measures ANOVA and adjusted using Bonferroni correction).

After a 28 days chronic study of *C. asiatica* (three doses, administered twice daily) on type 2 diabetic rats, 1000 mg/Kg dose showed significant reduction in serum glucose level (p < 0.05; Figure 4).

### Effect of *C. asiatica* on serum glucose after sucrose load

**Figure 5:**
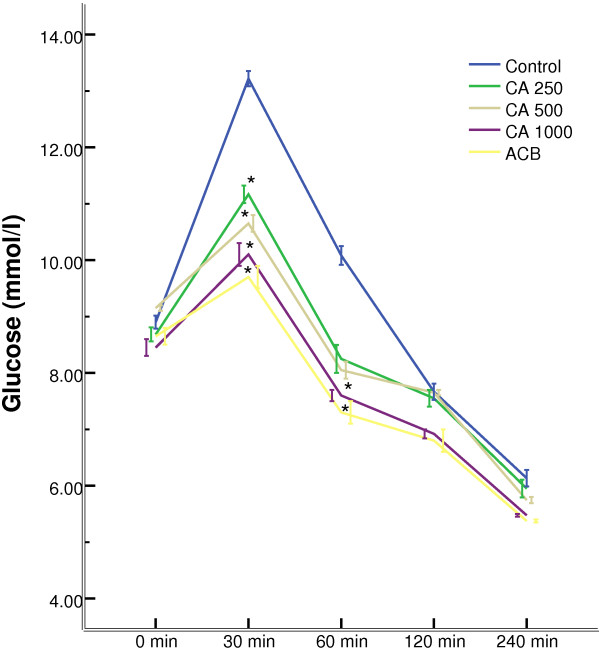
**Effects of ethanol extract of *C. asiatica *(CA) on serum glucose after the sucrose load in type 2 diabetic rats.** Rats were fasted for 20 h and administered orally with a sucrose solution (2.5 g/kg body weight) with or without ethanol extract of *C. asiatica* (250 mg/kg, 500 mg/kg, and 1000 mg/kg body weight) or Acarbose (ACB) (200 mg/Kg) or only water (control). Values are means and standard deviations represented by vertical bars (n = 8). Mean values marked with an asterisk (*) were significantly different from those of respective control rats (p < 0.05) (derived from repeated-measures ANOVA and adjusted using Bonferroni correction).

All the doses of *C. asiatica* showed a significant (p < 0.05) suppression of serum glucose level at 30 min compared to control, where peak serum glucose was observed after administration of sucrose load. 1000 mg/Kg dose of *C. asiatica* maintained this trend of suppression of glucose level at 60 min too (Figure 5).

### Effect of *C. asiatica* on intestinal glucose absorption

**Figure 6:**
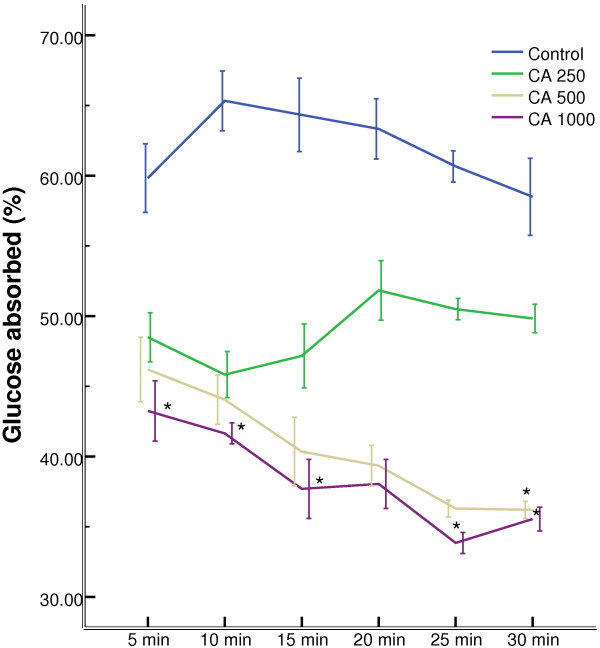
**Effects of ethanol extract of *C. asiatica *(CA) on intestinal glucose absorption in type 2 diabetic rats.** Rats were fasted for 36 h and the intestine was perfused with glucose (54 g/l) with (treated group) or without (control group) ethanol extract of *C. asiatica* (5 mg/ml, 10 mg/ml, and 20 mg/ml; each subject received 15 ml of perfusion). Values are means and standard deviations represented by vertical bars (n = 9). Mean values marked with an asterisk (*) were significantly different from those of respective control rats (p < 0.05) (derived from repeated-measures ANOVA and adjusted using Bonferroni correction).

1000 mg/Kg doses of *C. asiatica* extract, when perfusated with glucose, showed significant (p < 0.05) reduction in the percentage of glucose absorption during most of the perfusion period (Figure 6).

### Effect of *C. asiatica* on unabsorbed sucrose content in the gastrointestinal tract

**Figure 7:**
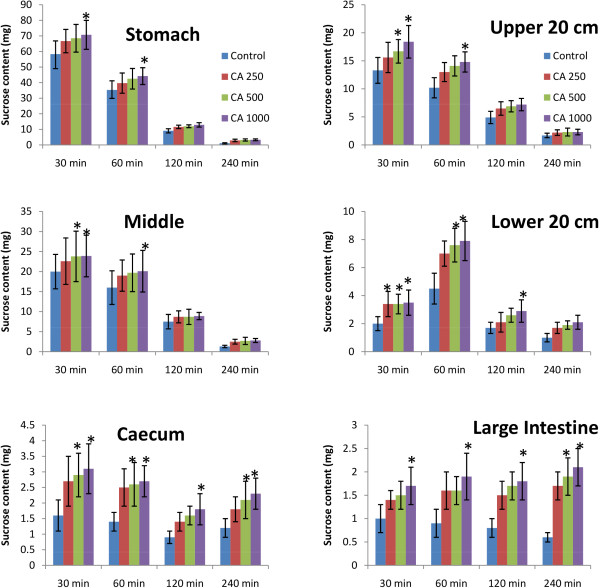
**Effects of ethanol extract of *C. asiatica *(CA) on gastrointestinal sucrose content after oral sucrose loading in type 2 diabetic rats.** Rats were fasted for 20 h before the oral administration of a sucrose solution (2.5 g/kg body weight) with (treated group) or without (control group) ethanol extract of *C. asiatica* (250 mg/kg, 500 mg/kg, and 1000 mg/kg body weight). Values are means and standard deviations represented by vertical bars (n = 8). Mean values marked with an asterisk (*) were significantly different from those of control rats (p < 0.05) (derived from repeated-measures ANOVA and adjusted using Bonferroni correction).

Upon oral administration of sucrose along with *C. asiatica* (1000 mg/Kg), significant amount of unabsorbed sucrose was remained in the stomach, upper, middle, and lower intestine at 30 min and 1 h. This amount of residual sucrose remained significant in caecum and large intestine till 4 h (p < 0.05; Figure 7).

### Effect of *C. asiatica* on gut motility and intestinal disaccharidase enzyme activity

**Figure 8:**
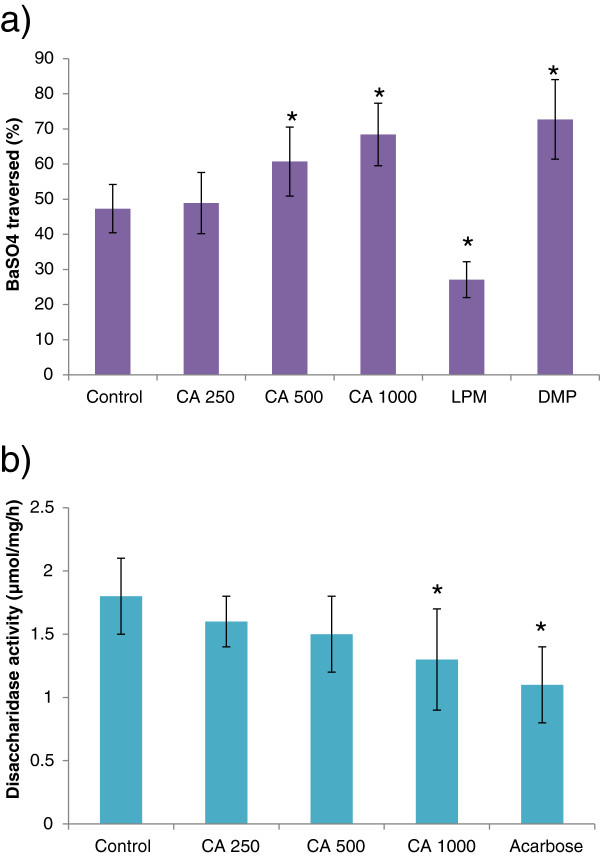
**Effects of ethanol extract of *C. asiatica *(CA) on a) gastrointestinal motility (by BaSO4 traversed) and b) intestinal disaccharidase activity in type 2 diabetic rats.** Rats were fasted for 20 h before the oral administration of ethanol extract of *C. asiatica* (250 mg/kg, 500 mg/kg, and 1000 mg/kg body weight) or water (control). Enzyme activity was determined and BaSO4 was administered at 60 min. Motility was measured over the following 15 min. Acarbose (ACB) (200 mg/Kg) and Loperamide (LPM) 5 mg/Kg) & Domperidone (DMP) (10 mg/Kg) were used as reference controls for disaccharidase activity test and gastrointestinal motility test respectively. Values are means and standard deviations represented by vertical bars (n = 12). Mean values marked with an asterisk (*) were significantly different from those of diabetic control rats (p < 0.05) (derived from repeated-measures ANOVA and adjusted using Bonferroni correction).

*C. asiatica* extract increased the gastrointestinal motility significantly (p < 0.05) at both 500 mg/Kg and 1000 mg/Kg doses. However, the extract showed significant (p > 0.05) inhibition of disaccharidase enzyme activity only at 1000 mg/Kg dose (Figure 8).

### Effect of *C. asiatica* on insulin secretion from isolated rat islets

*C. asiatica* extract, at any dose, did not induce any stimulatory activity on insulin secretion from isolated islets in the presence of both 3 mM and 11 mM glucose (Table 1).

**Table 1:** **Effect of ethanolic extract of CA ( *Centella asiatica *) on insulin secretion from isolated rat islets**

**Group**	**Insulin secretion (ng/mg islet protein)**	
	**Glucose: 3 mM**	**Glucose: 11 mM**
Control	2.99 (2.65-4.27)	5.41 (4.91-9.27)*
CA 20 μg/ml	2.78 (2.19-3.56)	5.03 (4.24-8.54)
CA 40 μg/ml	3.09 (2.43-3.82)	5.11 (4.39-7.98)
CA 80 μg/ml	2.85 (2.07-3.69)	5.24 (4.78-8.61)
Glibenclamide (10 μg/l)	5.89 (4.34-6.95)*	8.92 (7.67-9.86)*

Isolated rat islets were incubated for 60 min with ethanol extract of *Centella asiatica* (10, 20, and 30 mg/ml) in the presence of 3 or 11 mM glucose. Data are presented as median (range), n=5. Mann–Whitney U-test was used to evaluate statistical significance. *p < 0.05 compared with control (3 mM glucose without extract).

### Chronic effect of *C. asiatica* on Liver glycogen, organ weight, food habit, and serum lipid profile

**Figure 9:**
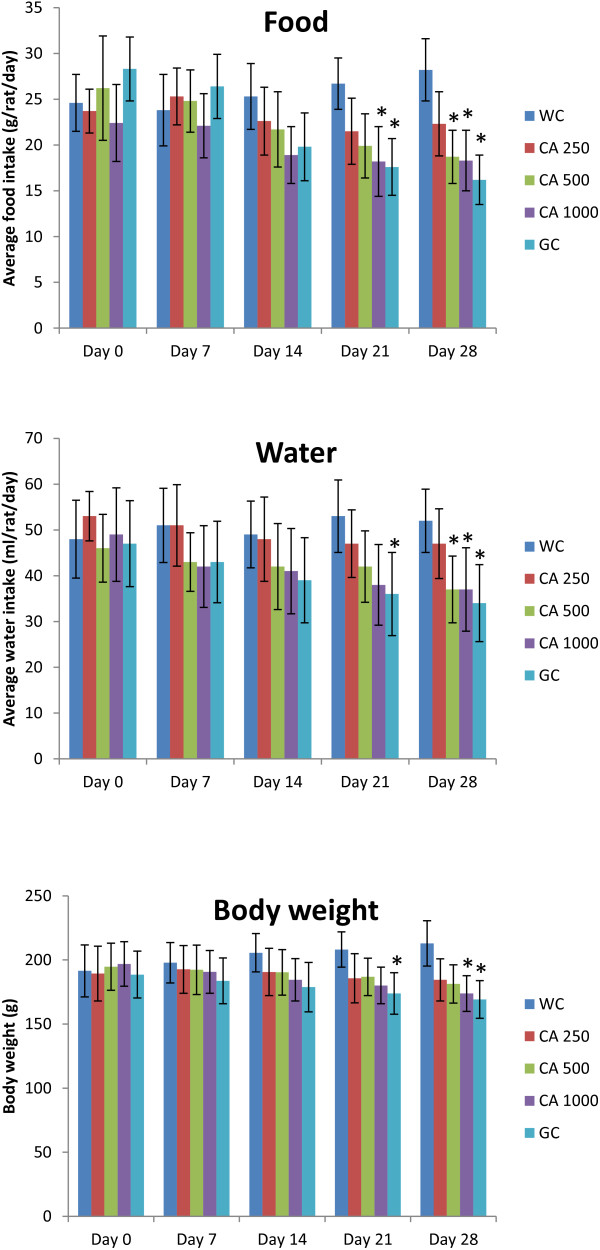
**Effects of ethanol extract of *C. asiatica *(CA) on body weight, average food intake, and average water intake in type 2 diabetic rats after 28 days of feeding.** Values are means and standard deviations represented by vertical bars (n = 10). Fasted rats were given ethanol extract of *C. asiatica* (250 mg/kg, 500 mg/kg, and 1000 mg/kg body weight) or Glibenclamide (GC) (0.5 mg/Kg) or only water (control) by oral administration for a period of 28 days. Mean values marked with an asterisk (*) were significantly different from those of respective control rats (p < 0.05) (derived from repeated-measures ANOVA and adjusted using Bonferroni correction).

**Figure 10:**
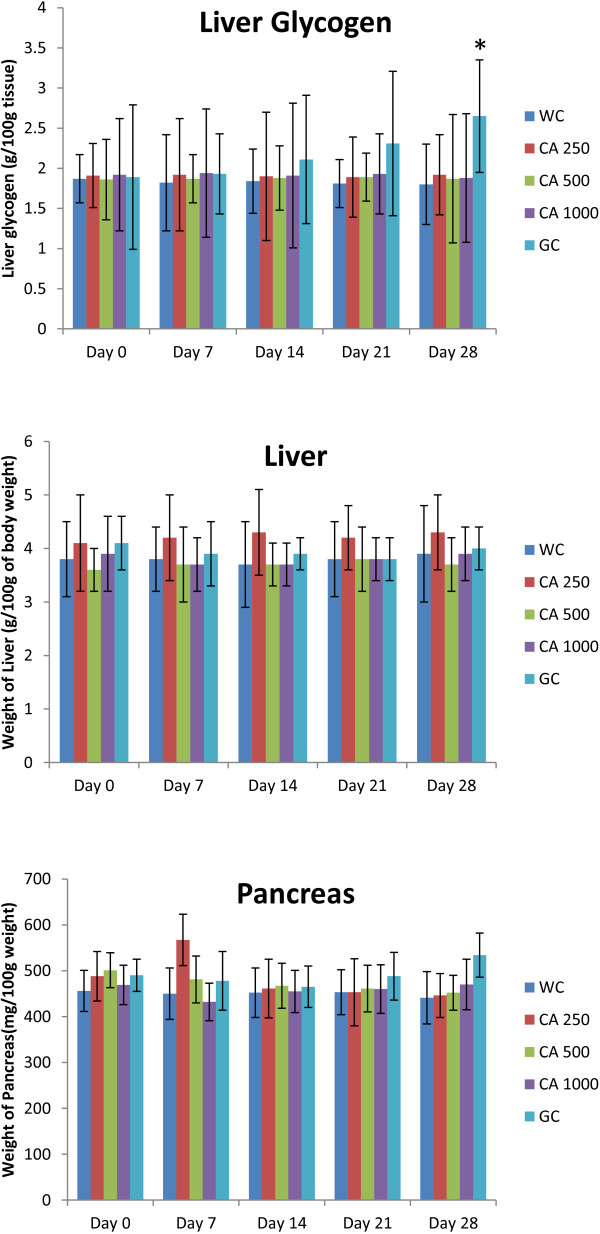
**Effects of ethanol extract of *C. asiatica *(CA) on liver glycogen, liver weight, and pancreas weight in type 2 diabetic rats after 28 days of feeding.** Values are means and standard deviations represented by vertical bars (n = 10). Fasted rats were given ethanol extract of *C. asiatica* (250 mg/kg, 500 mg/kg, and 1000 mg/kg body weight) or Glibenclamide (GC) (0.5 mg/Kg) or only water (water control, WC) by oral administration for a period of 28 days. Mean values marked with an asterisk (*) were significantly different from those of respective control rats (p < 0.05) (derived from repeated-measures ANOVA and adjusted using Bonferroni correction).

After the 28 days long study of C. asiatica (three doses, administered twice daily) on type 2 diabetic rats, no significant changes were seen in the liver glycogen content, liver weight, and pancreas weight (Figure 9). However, significant reductions in body weight, food intake, and water intake were observed at the end of the study (p<0.05; Figure 10).

**Figure 11:**
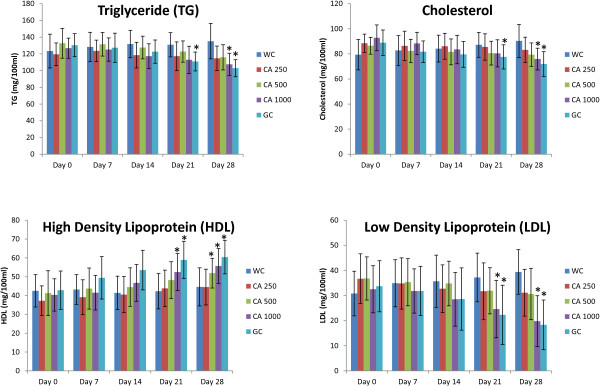
**Effects of ethanol extract of *C. asiatica *(CA) on serum lipid profile (TG, Cholesterol, HDL, and LDL) in type 2 diabetic rats after 28 days of feeding.** Values are means and standard deviations represented by vertical bars (n = 10). Fasted rats were given ethanol extract of *C. asiatica* (250 mg/kg, 500 mg/kg, and 1000 mg/kg body weight) or Glibenclamide (GC) (0.5 mg/Kg) or only water (control) by oral administration for a period of 28 days. Mean values marked with an asterisk (*) were significantly different from those of respective control rats (p < 0.05) (derived from repeated-measures ANOVA and adjusted using Bonferroni correction).

*C. asiatica* extract of 1000 mg/Kg dose improved serum lipid profile of type 2 diabetic rats after 28 days of twice daily oral feeding. It decreased the level of serum triglyceride, low density lipoprotein (LDL), and cholesterol significantly, which was comparable to the effect of reference drug Glibenclamide (p < 0.05; Figure 11). Moreover, the extract increased the level of high density lipoprotein (HDL) significantly (p < 0.05; Figure 11).

### Effect of *C. asiatica* powder on In vitro glucose dialysis retardation index (GDRI)

*C. asiatica* powder at different doses reduced the amount of glucose present in the dialysate. However, it was significant only for 1000 mg powder group; GDRI 45.8% and 48.54% at 30 min and 60 min respectively (p < 0.05; Table 2).

**Table 2:** Retarding effect of insoluble fibre of CA on the glucose movement (glucose dialysis retardation index)

**Treatment**	**Dialysis for 30 min**		**Dialysis for 60 min**	
	**Glucose in Dialysate(mmol/L)**	**Glucose dialysis retardation index (%)**	**Glucose in Dialysate(mmol/L)**	**Glucose dialysis retardation index (%)**
CA 250 mg	0.83 ± 0.19	30.25	1.15 ± 0.15	32.75
CA 500 mg	0.76 ± 0.12	35.29	1.09 ± 0.06	36.26
CA 1000 mg	0.65 ± 0.09*	45.38	0.88 ± 0.12*	48.54
CMC 1000 mg	0.62 ± 0.11*	42.86	0.83 ± 0.07*	51.46
Control	1.19 ± 0.21	0	1.71 ± 0.14	0

Data are presented as Mean ± SEM (n = 7). Glucose dialysis retardation index = control (100%) – fibre (% of control value). Mean values marked with an asterisk (*) were significantly different from those of respective control groups (p < 0.05) (derived from repeated-measures ANOVA and adjusted using Bonferroni correction).

### Effect of *C. asiatica* powder on α-amylase activity

The effect of *C. asiatica* powder on starch digestibility was determined by the alteration in the glucose concentration in the dialysate with time. There was no significant change, compared to control, in the glucose content at 10 min. However, the glucose content in the dialysate was significantly increased, compared to control, at 30 min, 1 h, and 2 h (p < 0.05; Table 3).

**Table 3:** Effect of insoluble fibre of CA on starch digestibility

**Treatment**	**Glucose in dialysate (μmol/l)**			
	**10 min**	**30 min**	**60 min**	**120 min**
CA 250 mg	1.72 ± 0.09	4.05 ± 0.15	13.6 ± 1.23	21.7 ± 4.10
CA 500 mg	1.81 ± 0.11	4.56 ± 0.42*	14.3 ± 2.32	23.50 ± 5.20*
CA 1000 mg	1.85 ± 0.15	4.88 ± 0.50*	15.89 ± 2.11*	26.45 ± 4.83*
CMC 1000 mg	1.91 ± 0.21	4.94 ± 0.34*	16.09 ± 2.31*	26.57 ± 5.34*
Control	1.52 ± 0.06	3.87 ± 0.12	12.5 ± 1.23	19.5 ± 3.73

Data are presented as Mean ± SEM (n = 4). Values represent the glucose concentration (μmol) in dialysate. Mean values marked with an asterisk (*) were significantly different from those of respective control groups (p < 0.05) (derived from repeated-measures ANOVA and adjusted using Bonferroni correction).

### Effect of *C. asiatica* powder on In vitro glucose adsorption capacity

*C. asiatica* powder showed high capacity of glucose adsorption in the presence of different levels of glucose in the solution. This activity of glucose adsorption was found to persist from higher level of glucose to even very low level of glucose present in the solution (Table 4).

**Table 4:** Glucose adsorption capacity of insoluble fibre of CA in different concentrations of glucose

**Treatment**	**Glucose bound (mmol/g)**				
	**5 mmol/l**	**10 mmol/l**	**50 mmol/l**	**100 mmol/l**	**200 mmol/l**
CA 250 mg	0.03 ± 0.01^a^	0.91 ± 0.11^a^	4.88 ± 0.93^a^	9.23 ± 1.02^a^	19.43 ± 3.37^a^
CA 500 mg	0.06 ± 0.01^b^	1.87 ± 0.43^b^	6.78 ± 2.10^b^	13.89 ± 3.67^b^	23.88 ± 4.87^b^
CA 1000 mg	0.08 ± 0.02^c^	2.67 ± 0.61^c^	8.93 ± 1.98^c^	16.75 ± 4.12^c^	29.94 ± 7.50^c^
CMC 1000 mg	0.08 ± 0.01^c^	2.71 ± 0.45^c^	9.32 ± 2.09^c^	17.81 ± 3.78^c^	30.97 ± 6.88^c^

Data are presented as Mean ± SEM (n = 4). Data represent the millimoles of glucose bound by each gram of the CA extract at different glucose concentrations (5-200mmol/l). Glucose bound = (glucose concentration of original solution - glucose concentration when the adsorption reached equilibrium) × volume of solution ÷ weight of dietary fibre. Mean values in the same column marked with different letters (^a^, ^b^, and ^c^) were significantly different at p < 0.05 (Values which were found to be statistically similar to the positive control were denoted as “^c^”; they present strong fibre binding. Values denoted by “^a^” and “^b^” were statistically different from both the positive control and themselves; these values represent non significant fibre binding) (derived from repeated-measures ANOVA and adjusted using Bonferroni correction).

## Discussion

*C. asiatica* is a plant, native to the Indian Subcontinent, continental Asia, Australia and Papua New Guinea [22]. It is indigenously used to treat a wide range of pathological conditions including diabetes, which provides a solid ground for our current study [8-10]. Additionally, unpublished, preliminary screening data, of this plant, showed highly promising hypoglycemic activity. However, there are no reported studies on tissue level mechanism of action of *C. asiatica*. Studies have established that hyperglycemic states, during diabetes, is the initiator of diabetic tissue damage [23]. Cells damaged by hyperglycemia cannot maintain a constant internal glucose concentration, which results in acutely altered cellular metabolism and long-term changes in cellular macromolecular content [24-26]. Postprandial glucose spike causes perturbation in endothelial cell function [27,28], and increased blood coagulation [29]. Hyperglycemic states also increases products of glycosylation, which has a significant influence in development of diabetic induced vascular disease [30]. Therefore, management of hyperglycemic states is an important method of diabetes control. The basic mechanism of actions of commonly used diabetic drugs are, enhanced insulin secretion, enhanced sensitivity to insulin, improved peripheral glucose utilization, inhibition of glucose absorption, and inhibition of carbohydrate digestion [31]. In our current study, we have employed techniques, which will suggest one or more of the aforementioned modes of action.

Altered lipid metabolism is a hallmark of type 2 diabetes induced dyslipidemia, which is characterized by reduced HDL and increased LDL, triglycerides, and total cholesterol [32]. Hyperglycemia and altered lipid status, in tandem, poses a significant threat of cardiovascular complications in diabetic patients. Unattended hyperlipidemia might give rise to both micro and macro vascular complications in type 2 diabetic patients [33]. Therefore, alleviated lipid profile in patients might improve diabetes induced secondary complications. Type 2 diabetic rats treated with 1000 mg/kg of *C. asiatica* extract showed marked improvement in serum lipid profile at the end of our 28 days study period.

Type 2 diabetic patients have a higher incidence of obesity. It is also characterized by polyphagia and polydipsia [34]. By the end of our study period, *C. asiatica* treated Type 2 diabetic rats returned to a normal body weight and exhibited food and water intake comparable to healthy rats.

Studies have shown that blood glucose level in the upper normal range is a probable risk factor for cardiovascular disease, a condition, chronic in Type 2 diabetic patients [35]. In our studies, fasting blood glucose remained unaffected in Type 2 diabetic rats in all groups apart from the “Glibenclamide” control group. In glucose tolerance test, the peak glucose concentration after glucose challenge in *C. asiatica* treated group at 1000 mg/kg dose did not increase as sharply as the control group. The glucose concentration was significantly lower than the 250, 500 mg/kg treated group or the control group. Two of the above findings reinforces previous claims about *C. asiatica* having anti-hyperglycemic activity without inducing hypoglycemia [8,9]. Therefore, it might be devoid of one of the key flaws in many of the currently prescribed anti-diabetic medications [36].

To further ascertain, mechanism of anti-diabetic action, we measured the plasma insulin level of the test animals and found no significant increase in insulin secretion on *C. asiatica* administration, at all doses. This preliminary finding was further strengthened by a similar lack of activity shown by *C. asiatica* on isolated rat islets. Furthermore, liver glycogen content remained unaltered with respect to control group. Therefore, increased insulin secretion or increased sensitivity to insulin action can both be ruled out. It is to be noted that, wet mass of both liver and pancreas in control and treated groups did not significantly differ. Regeneration of pancreatic β-cell mass and enhanced glucose utilization can be preliminary ruled out [37].

An in situ intestinal perfusion of the GI tract shows marked reduction in glucose absorption. In BaSO_4_ GI motility assay, intestinal motility was found to be significantly higher. Numerous published results have shown the ability of complex carbohydrate, high molecular weight, and viscous, soluble dietary fibers, to retard glucose absorption [38-40]. *C. asiatica* has been said to contain oligosaccharide centellose, resin and large amounts of insoluble dietary fibers [41]. Dietary fibers often provide a greater barrier to diffusion caused due to its high viscosity and ability to bind to glucose [38]. Dietary fibers are capable of significantly reducing the transit time in GI Tract of ingested food [42]. Reduced transit time can be translated as lesser time available for di-and polysaccharides in the meal to be digested and absorbed [43]. It logically follows a lower glucose peak concentration after the meal.

Six Segment test showed significantly higher amount of sucrose in stomach, upper, middle and lower intestine in *C. asiatica* administered groups. The latter three part of GI are most important for absorption of nutrients including sugars [44]. Disaccharides in its own form does not get absorbed due to lack to sucrose carriers, as carriers monosaccharides only are present in the GI tract [45]. Therefore, it is imperative that disaccharides get converted to monosaccharides first for absorption. Higher sucrose content in the GI Tract clearly reflects a reduced sucrose digestion throughout the GI Tract. This in turn, is shown by a significantly higher concentration of sucrose reaching the large intestine and caecum, which eventually remains unabsorbed and egested with faeces.

In the intestinal disaccharidase activity and the α-amylase activity assay, *C. asiatica* was shown to have reduced the catabolism of sucrose and starch respectively. Since complex carbohydrates and disaccharides have first to be broken down into simpler monosaccharides [45], it follows that any inhibition of this catabolic process would retard sugar absorption, which would in turn, be shown as a lower glycemic peak. However, the precise mechanism of this inhibitory action remains to be studied.

In-vitro studies involving fiber binding assays clearly demonstrated glucose being bound by dietary fibers available in *C. asiatica* even at very low glucose concentrations. Glucose are carried by specific transport proteins [45]. Bound glucose is probably incapable of fitting the active site of these transport proteins. This fully validates our initial findings in the gut perfusion experiments, which too showed a hindrance in glucose absorption. This now can be fully attributed to glucose-fiber binding in *Centella asiatica* whole plant powder.

Further research is underway, in our labs, for identifying the active molecules responsible for inhibiting α-amylase and disaccharidase. We also intend to study the protein transporters, most affected by the active compound, via radio-ligand binding assay. In conclusion, we report absence of enhanced insulin secretion in *C. asiatica* treated animal groups. No significant improvement was seen in liver glycogen deposition either. Findings in previous studies reporting anti-hyperglycemic activity in *C. asiatica*, can be probably attributed to reduced carbohydrate breakdown, glucose-fiber binding, thus, and overall reduced glucose absorption through the GI tract. Furthermore, on chronic intake, it has ability to lower harmful LDL and Cholesterol in animal models while elevating the beneficial HDL. *C. asiatica* calls for further attention from the scientific community to further elucidate its activity and establish its safety profile.

## Conclusions

Our studies confirm the previous findings showing anti-hyperglycemic action of *C. Asiatica.* Additionally, we have elucidated that *C. Asiatica* is capable of inhibiting absorption of glucose both by inhibition of intestinal disaccharidase enzymes and α-amylase and by glucose-fibre binding. On chronic intake, it has lowers serum LDL and Cholesterol and elevates the HDL. Therefore, its traditional use, as mentioned above is justified and calls for further research, to optimize its anti-diabetic activity.

## Competing interests

This study was funded by a “Graduate Research Grant” of North South University.

## Authors’ contributions

AUK: Conducted experiments, carried out Data Analysis, revised the preliminary manuscript. MBS: Designed and established in-vitro protocols, conducted experiments, wrote the manuscript. NMD’C: Carried out experiments, wrote the manuscript, and revised the manuscript. FA: Carried out the in-vitro tests and phytochemical analysis. She was also responsible for preparation of the extract. AA: Carried out the initial screening tests and toxicological studies on the used extract. He was responsible for preparation of the extract. JMAH: Provided overall supervision and coordinated all experimental activities, initially approved the experimental protocols, revised manuscript, carried out experiments. All authors read and approved the final manuscript.

## Pre-publication history

The pre-publication history for this paper can be accessed here:

http://www.biomedcentral.com/1472-6882/14/31/prepub
